# Frequency, dynamics, and duration of faecal shedding in SARS-CoV-2-infected individuals, a scoping review

**DOI:** 10.1017/S0950268826101241

**Published:** 2026-03-24

**Authors:** Susan Abunijela, Timo Greiner, Walter Haas, Romy Kerber, Peter Pütz, Alexander Schattschneider, Jakob Schumacher, Udo Buchholz

**Affiliations:** Infectious Disease Epidemiology, Robert Koch-Institut, Germany

**Keywords:** COVID-19, SARS-CoV-2, Faeces, Virus Shedding, Wastewater

## Abstract

To estimate illness incidence or prevalence from wastewater data, modelling approaches may benefit from incorporating faecal shedding parameters. We systematically searched PubMed and a public repository on shedding data and included 33 studies that met at least one of our objectives. Among 32 studies, the proportion of SARS-CoV-2-infected individuals with detectable virus in stool ranged from 18 to 100%, with a pooled estimate of 54% (95% CI: 52–56%). Stratification by four clinical severity categories, ranging from asymptomatic to critically ill, showed no significant differences among categories (p-value = 0.49). The proportion of individuals with detectable SARS-CoV-2 RNA in stool was higher in children (61%) than in adults (53%; p-value = 0.02). In half of the individuals who initially shed the virus in stool, it remained detectable for an estimated 22 days post-symptom onset. Three studies documented viral load kinetics, indicating a peak between days 3 and 9. Twenty-five studies reported maximum shedding durations ranging from 2 to 12 weeks. Our review summarizes the frequency, dynamics, and duration of SARS-CoV-2 shedding in stool and may serve as a valuable foundation for modelling efforts involving faecal shedding indicators.

## Introduction

SARS-CoV-2 transmission occurs primarily through respiratory pathways [1], while other transmission routes are of minor importance [2]. Nevertheless, many SARS-CoV-2-infected individuals release virus or viral fragments in their stool, enabling reliable detection in wastewater [3, 4]. This has led to the widespread establishment of monitoring SARS-CoV-2 through wastewater surveillance systems.

Several studies have used faecal shedding data in combination with other information to predict SARS-CoV-2 incidence or prevalence from wastewater data [5, 6]. These models rely on faecal shedding parameters to link the number of infected individuals with the amount of viral RNA detectable in wastewater. However, many studies have made assumptions or used shedding parameters from individual papers, such as the proportion of infected individuals who shed the virus, the timing of peak shedding, and the duration of shedding data derived from other clinical data [7]. Therefore, it is crucial to make shedding parameters available that integrate the current knowledge to refine wastewater-based models.

The average shedding of SARS-CoV-2 RNA in stool can be formally quantified using the following parameters:

1. **Proportion of patients with detectable viral RNA in stool** – the fraction of diagnosed SARS-CoV-2-infected individuals who have viral RNA detectable in stool.
2. **Decline over time** – how fast does the positivity rate (PR; proportion positive) of patients with initially detectable viral RNA in stool decline over time and after symptom onset.
3. **Viral load kinetics** – the timing of the magnitude of viral RNA in stool; and: what is the temporal sequence of the shedding peak in stool and respiratory samples respectively?
4. **Shedding duration** – the maximum duration of faecal viral shedding time (VST).

Several reviews have addressed some of these questions and parameters [3, 8–12]. However, many reviews did not apply standardized criteria for key factors (e.g., requirement of a systematic approach to stool sampling, considering the duration of follow-up, etc.). Additionally, some did not address key limitations of the underlying studies. Most reviews have made statements about the proportion of patients (or persons with SARS-CoV-2 infections) of whom viral RNA can be detected in the stool [3, 9–12], and the maximum duration of virus in stool [3, 11, 13–15]. Two studies tried to characterize the dynamic aspects [6, 16], and none have covered all aspects named above. In addition, the use of specific population subsets or differing methodologies of the underlying studies has led to inconsistent findings, particularly regarding how long individuals shed viral RNA in stool.

Our review aims to synthesize available evidence on the following parameters:

1. Proportion of persons infected with SARS-CoV-2 who shed viral RNA in the stool.
2. Among individuals with faecal shedding:
  - Decline of PR over time.
  - Dynamic course of viral RNA in stool over time.
3. Maximum duration of faecal viral shedding.

## Methods

### Information sources and search strategy

We searched PubMed using a combination of MeSH terms and keywords linking the three categories: population (SARS-CoV-2-infected individuals), sample material (stool), and parameters (including positivity rate, kinetics, and viral load) using the AND operator (Appendix; Table A1). Additionally, we screened a public repository on shedding data [17] to identify further relevant studies.

### Inclusion and exclusion criteria

We included studies in which SARS-CoV-2 infections were confirmed via positive respiratory swabs prior to stool sample collection. Only studies that began collecting stool samples during the first week after symptom onset. Furthermore, we selected studies that collected at least three stool samples for the majority of participants.

To calculate the proportion of SARS-CoV-2-infected individuals in whom SARS-CoV-2 was also detected in stool (objective 1), only studies that reported this indicator based on the number of participants, rather than the number of samples, were included. To estimate the proportion of SARS-CoV-2-infected individuals with positive stool (trend of the PR) over time (objective 2a), studies that involved SARS-CoV-2-infected individuals who regularly provided stool samples over a period of time, were included. To calculate the shedding dynamic (of the PR, objective 2a, or viral load, objective 2b), we included only studies that specified the day of sample collection with respect to symptom onset (and not, e.g., with respect to hospital admission), as well as studies that incorporated SARS-CoV-2-negative stool samples in their proportion of patients with faecal shedding or in their analysis of viral shedding kinetics. To calculate the maximum VST of the detectable virus in stool samples (objective 3), only studies that provided duration of VST, with a minimum follow-up period (at least 2 weeks), in time after symptom onset were included.

We excluded studies with unclear methodologies, such as studies that lacked information on the number of stool samples taken per patient, as well as studies with vague patient selection criteria. Additionally, during the screening process, studies focusing on animals and those that were not written in English or German were disregarded.

### Selection of studies

We imported the identified studies into EndNote for de-duplication and transferred the remaining studies to Microsoft Excel (Microsoft Corporation, Redmond, WA, USA). One author (S.A.) reviewed the titles and abstracts of the identified studies according to the inclusion and exclusion criteria and classified them as preliminarily included, excluded, or uncertain. Studies initially classified as ‘uncertain’ were discussed with a second author (U.B.) until all conflicts were resolved. Full texts were then carefully examined by (S.A.) and again reclassified as included, excluded, or uncertain, and remaining uncertainties were resolved through discussion with (U.B.).

### Data extraction

For each included study, we systematically extracted the following details: author(s), journal, publication year, title, main findings, including the proportion of infected individuals with shedding, PR over time, shedding kinetics, and duration of shedding. One reviewer (S.A.) extracted the data while a second reviewer (U.B.) checked it for accuracy to ensure consistency and reliability.

### Calculation of key parameters

For the three study objectives, we calculated the proportion of infected individuals who also shed virus in the stool, the PR over time, the kinetics of the viral load, and the VST. Details and the related statistical analysis are summarized in Table 1.

**Table 1.** Summary of the calculation of key parameters

Key parameters of objectives	Calculation method
(1) Proportion of SARS-CoV–2-Infected individuals shedding viral RNA in stool	The pooled proportion of individuals infected with SARS-CoV–2 with detectable viral RNA in stool was calculated using the following formula: 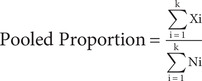 Where: Xi = number of SARS-CoV–2-infected individuals with detectable viral RNA in stool in study i, Ni = total number of SARS-CoV–2-infected individuals in study i, k = total number of studies included in the analysis. We used the Exact (Clopper–Pearson) method to calculate 95% confidence intervals (CIs) for the overall detection rate. We then examined the association between clinical severity and stool SARS-CoV–2 RNA proportion using categorical stratification across four groups: asymptomatic individuals, symptomatic non-hospitalized individuals, hospitalized individuals with mild to moderate symptoms, and hospitalized individuals with severe illness. This analysis was conducted based on the studies from which we were able to extract the relevant data. The chi-square test for independence was used to assess whether detection rates differed significantly across severity levels. To evaluate potential trends across these ordered categories, we applied the Cochran-Armitage test for trend. Subsequently, we stratified the data by age group (children, adults), where available, and performed the same calculations.
(2a) Viral shedding dynamics: positivity rate over time	To analyse the temporal dynamics of SARS-CoV–2 RNA detection in stool, we compiled and harmonized individual-level data from multiple studies. Each dataset included the number of positive and total stool samples collected per day since symptom onset. The daily PR was computed as the number of positive patients divided by the total number tested per day, per study. We calculated pooled daily estimates by aggregating the number of positive and total samples across all studies and computed the weighted PR for each day after symptom onset. To characterize the decline of SARS-CoV–2 RNA detection in stool over time, we applied a Weibull decay model to the pooled daily PR. The Weibull model was selected for its ability to represent biological processes with non-linear decay function, accommodating both rapid and gradual decreases in detectability over time, such as viral shedding, while remaining interpretable, following a parametric form: PR(t) = exp. (− (t / λ) ^ k) where PR(t) is the positivity rate on day t, λ is the scale parameter, and k is the shape parameter. The model was used to predict positivity rates up to the first day the pooled rate dropped below 5%. A 95% CI for the predicted curve was computed from the bootstrap parameter sets. We plotted the data points until the 21st day after symptom onset because that is the time period with reliable data. The time range from day 21 onwards was defined as extrapolated since no further observed data were included in model fitting beyond that point.
(2b) Viral shedding dynamics: Kinetics of viral load (amount of SARS-CoV–2 copies per sample or weight of sample)	We documented the quantities of gene copies per sample or per milligram of sample for each day following symptom onset. Due to the large differences in measurements across the included studies, we normalized the values in each study’s data series to the highest value within that respective study. For instance, if in one study the highest value for a sample is 40,000 gene copies and another sample yielded 20,000 gene copies, the first sample would be set as 100%, while the second would be 50%. This approach allows for the visualization of the kinetic curves of several studies in one figure.
(3) Maximum duration of faecal viral shedding (VST)	Duration of viral shedding can be defined in different ways. One way is to model the PR over time and provide the estimated value of the 95% percentile [19]. Other authors state the ‘viral shedding time’ as the maximum time of any patient with detectable virus after symptom onset [10, 11]. We, in this review, used the latter definition.

## Results

**Figure 1.**
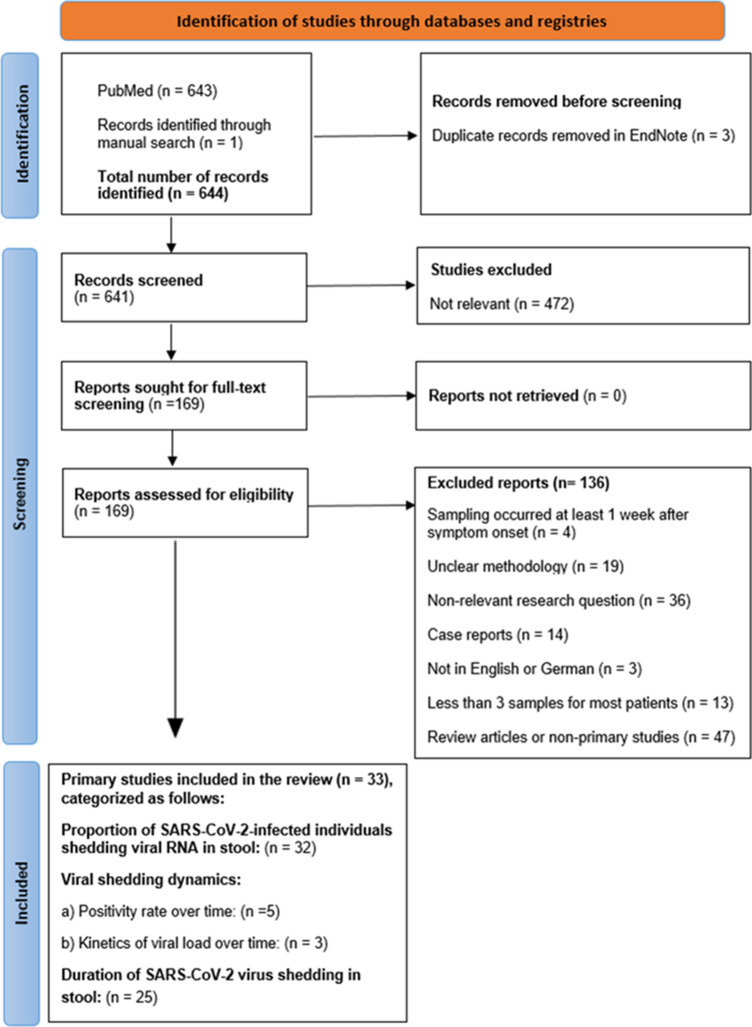
PRISMA flow chart depicting the screening process for filtering suitable primary studies for the review.

The PRISMA flow chart [18] outlines how included studies are selected, as illustrated in Figure 1. Out of the 644 identified studies, 33 primary studies were included in this review. Most studies were conducted between January 2020 and August 2021, with two later studies in February 2021–January 2022 [19] and 2023 [20]. Participants of studies exclusively conducted in 2020 were not vaccinated, or vaccination was not reported. Similarly, participants of studies partially or entirely conducted in 2021, that is, when vaccines had become increasingly available, were primarily not vaccinated or vaccination was not reported, with few exceptions (Appendix; Table A2). Notably, the study that was conducted in 2023 involved fully vaccinated participants, mostly without a booster, with a 100% detection rate of SARS-CoV-2 RNA in stool [20].

### Objective 1: Proportion of persons infected with SARS-CoV2 who shed viral RNA in the stool

**Figure 2.**
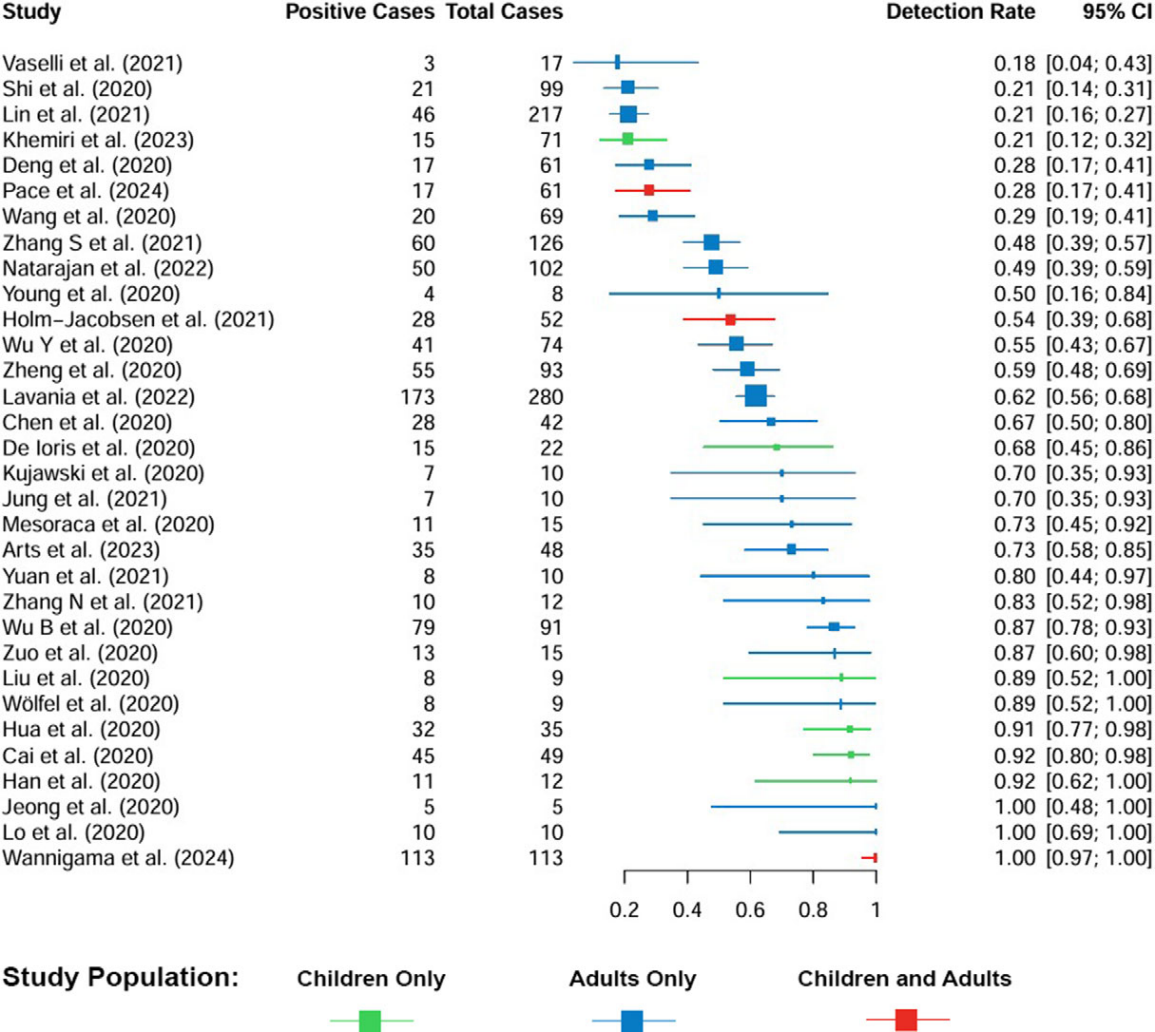
Proportion of individuals with SARS-CoV-2 infection (total cases) in whom viral RNA was detected in stool samples (positive cases). Squares represent the effect estimate for each study (the proportion of SARS-CoV-2-infected individuals with faecal shedding of viral RNA), with larger squares indicating larger sample sizes. Note: This figure provides a descriptive summary of detection rates across studies without meta-analytical models. The Exact (Clopper–Pearson) method was used to calculate 95% CIs.

**Figure 3.**
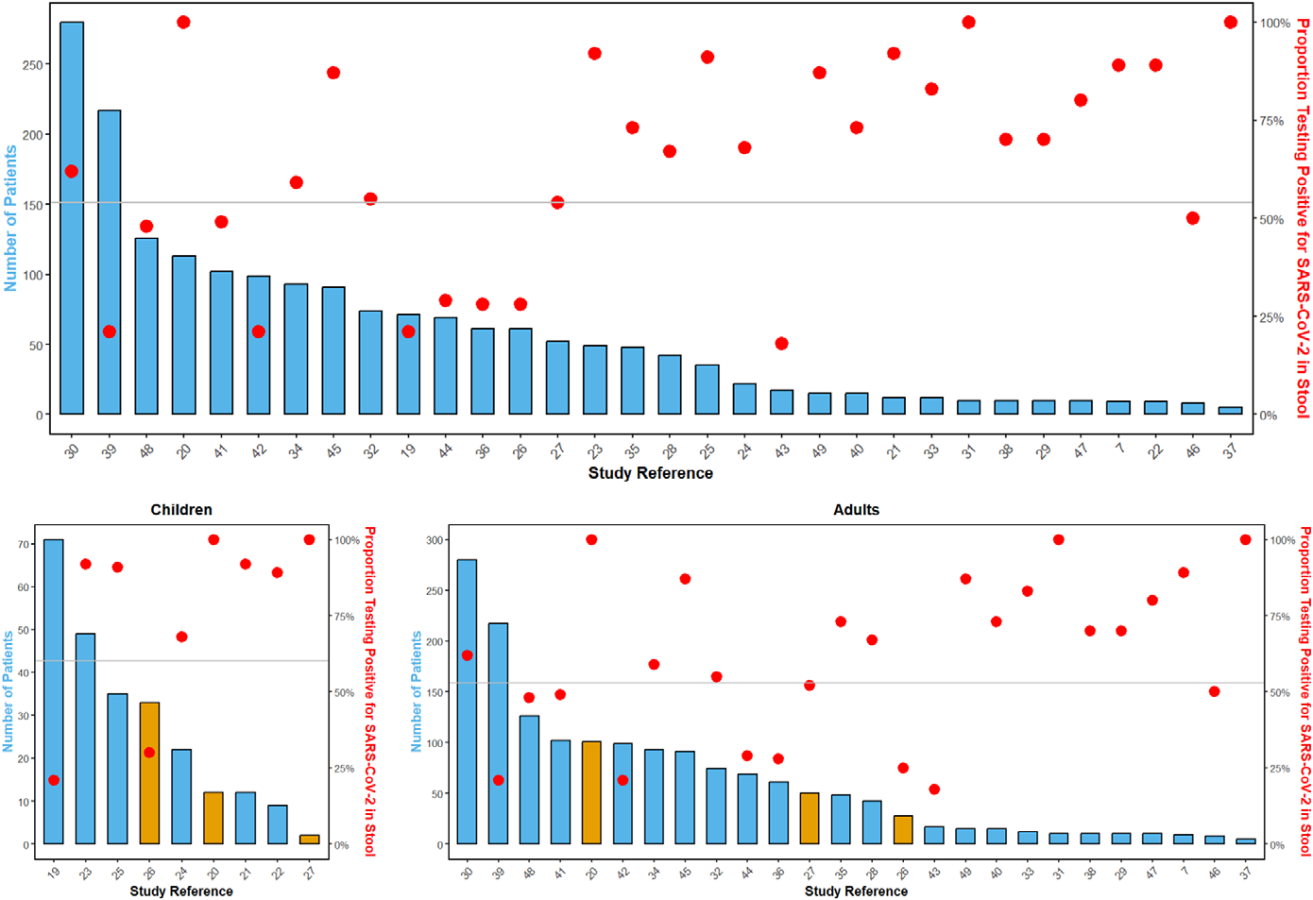
Proportion of patients with detectable SARS-CoV-2 in stool across three categories: all studies, studies only in children (Of these, six targeted only children [19, [21]–25] and three studies included children and adults [20, [26], 27]), and studies in adults (23 of these addressed adults only [7, [28]–49]). The x-axis displays the study reference. The left y-axis, corresponding to the bars, represents the quantity of patients per study, and the right y-axis, corresponding to the red dots, indicates the proportion of SARS-CoV-2-infected individuals testing positive for the virus in stool. Orange bars in the children’s and adults’ panels mark studies that involved both children and adults and contributed stratified data for the respective age category. The pooled proportion is displayed by the horizontal grey line: 54% for all studies (top panel), and 61% and 53% for the children’s and adults’ panels respectively.

Out of the 33 analysed studies, 32 provided data on the proportion of SARS-CoV-2-infected individuals with detectable viral RNA in their stool (Figure 2). The studies included between 5 and 280 participants (median = 45). Furthermore, the proportion of SARS-CoV-2-infected persons with faecal shedding varied from 18 to 100%. The mean proportion across studies was 64% (standard deviation = 26%), reflecting substantial heterogeneity. Because smaller studies tended to report higher proportions, we also calculated a pooled proportion of 54% (995 of 1847 patients; 95% CI = 52–56%, Figure 3).

Pooled proportions were higher in children (61%) compared with adults (53%) (p-value = 0.02), indicating a significant difference in detection rates between the age groups. Detection rates stratified by clinical severity ranged from 35% in asymptomatic individuals to 42% in those with severe illness, without significant differences (p-value = 0.49; p-value for trend = 0.16). Although most studies contribute to both subgroup analyses, stratification by age and by clinical manifestation highlights different aspects of the population. Age-based stratification includes all reported individuals, regardless of symptom status or illness severity. Detailed subgroup results are shown in Table 2 and Figure 3.

**Table 2.** Proportion of SARS-CoV-2-infected individuals shedding viral RNA in stool by subgroup

Stratification category		No. of studies	Range of participants	Detection rate range	Detection rate (positive cases/total cases)	(95% CI)
Age		32				
	Children	9	2–71	21%–100%	61% (150/245)	55%–67%
	Adults	26	5–280	18%–100%	53% (845/1602)	50%–55%
Clinical disease manifestation		24				
	Asymptomatic	4	2–37	18%–100%	35% (49/139)	27%–44%
	Symptomatic (non-hospitalized)	6	1–102	20%–100%	39% (150/383)	34%–44%
	Hospitalized (mild to moderate cases)	17	1–121	28%–100%	39% (240/613)	35%–43%
	Hospitalized (severe cases)	10	2–174	20%–100%	42% (169/399)	37%–47%

### Objective 2a: Viral shedding dynamics: Positivity rate over time

**Figure 4.**
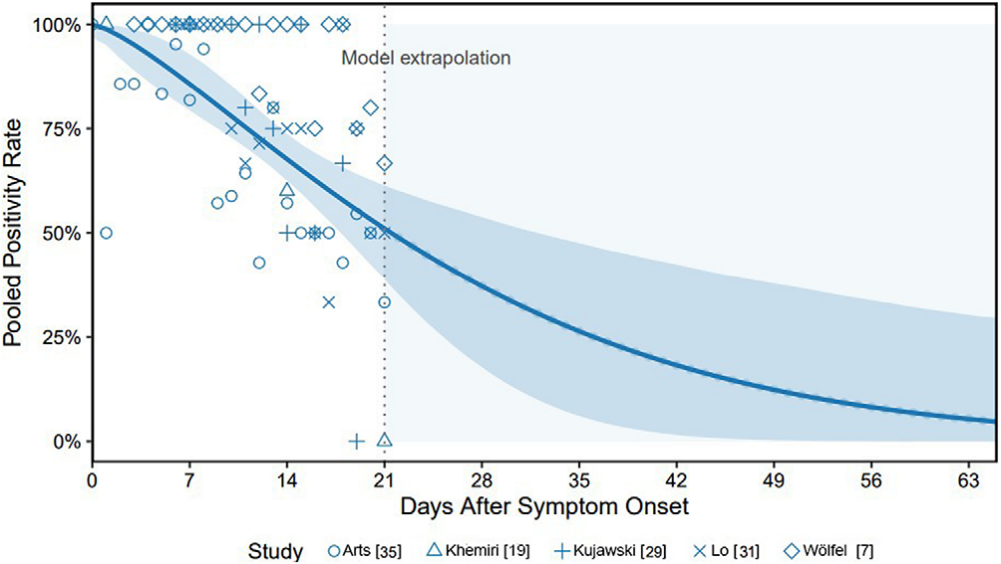
Pooled proportion of SARS-CoV-2 RNA-positive stool samples by day since symptom onset across five studies [7, [19], [29], [31], 35]. X-axis shows days after symptom onset; Y-axis shows pooled PR. The observed PR from five studies are displayed as blue markers with distinct shapes. The solid blue line represents the fitted Weibull decay curve to the daily weighted PR computed from pooled data. The shaded blue ribbon depicts the 95% CI derived from 5,000 bootstrap replicates. A vertical dashed line at day 21 marks the end of empirical data; the pale-blue shaded area to the right denotes the extrapolation range.

For this analysis, we included data from five studies (466 stool samples through day 21; Figure 4). The pooled PR exceeded 75% in the first week. According to the trend analysis, 50% of the people who initially excreted SARS-CoV-2, still excreted SARS-CoV-2-positive stool on day 22 after symptom onset, that is, corresponding to a period of approximately three weeks. The Weibull model predicted a decline below 5% by day 65. Study-level variability was observed, particularly in early and late time points, but the overall pooled trend was robust.

### Objective 2b: Viral shedding dynamics: Kinetics of viral load over time

**Figure 5.**
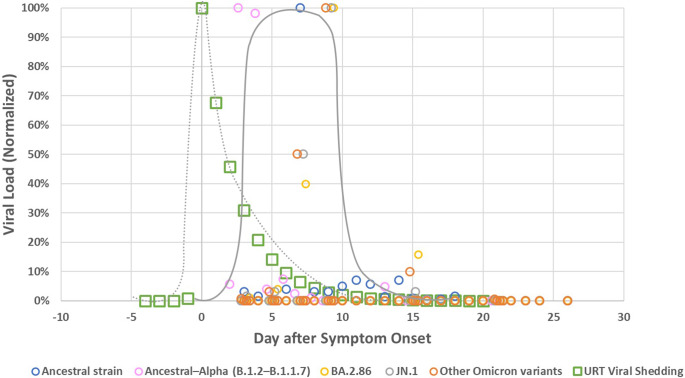
Normalized viral load in stool (expressed as a percentage of the maximum value within each data series in stool and upper respiratory tract (URT) samples from individuals infected with SARS-CoV-2, plotted by days after symptom onset. The circles represent stool viral load data derived from three studies [7, [20], 35]. Blue circles correspond to study [7] and reflect the ancestral strain. Pink circles represent data from study [35] and include both ancestral and Alpha variants. Yellow, grey, and orange circles originate from the study [20] and represent different Omicron variants. Green squares show URT viral data for comparison [50]. Solid and dashed grey lines represent non-parametric approximations of faecal and URT viral kinetics respectively. These lines were included for visual comparison only and were not derived from formal statistical fitting. The y-axis presents normalized viral load values on a linear scale.

We extracted the information regarding the amount of the viral load in stool from only three studies that provided robust data [7, [20], 35] (Table 3). Figure 5 shows normalized viral kinetics by days after symptom onset. For reference, viral load kinetics in the upper respiratory tract (URT) from a separate study are also displayed (green squares) [50], typically preceding the faecal kinetic curve by 3–10 days. Sharp peaks are noticed across the different SARS-CoV-2 variants included in each study, with peaks occurring at day 3 [35], 7 [7], and 9 [20] respectively.

**Table 3.** Comparison of Peak SARS-CoV-2 Viral Load Kinetics Over Time and Variants: Insights from Three Studies; SO = symptom onset

Study name	Number of patients included	Variant covered	Time of study or circulation of the variant in Germany	Peak Viral Load (Day after SO)	Unit of measurement	Notes
Wölfel et al. [7]	9	Ancestral strain	Jan 2020 – Mar 2021	Days 5–10 after SO	RNA copies per g of stool; peak concentration = 1,000,000 RNA gene copies per gram wet stool (gc/g-wet)	Conducted at the beginning of the pandemic in Germany.
Arts et al. [35]	48	Ancestral strain B.1.2, Alpha variant (B.1.1.7)	Dec 2020 – Mar 2021	Days 3–4 after SO	RNA copies per mg of dry matter; peak concentration = 3,557,620 gc/g-wet	Conducted in the USA during the dominance of ancestral SARS-CoV–2 lineages, including B.1.2, and the early emergence of the Alpha variant (B.1.1.7).
Wannigama et al. [20]	113	Omicron variants (BA.2.86, JN.1, others)	Aug 2022 – Dec 2023 BA.2.86 (from August 2022 to January 2023), Omicron JN.1 (from January 2023 to June 2023), and other summarized omicron variants that circulated from January 2023 to the end of 2023.	Days 5–10 after SO	RNA copies per mg of stool; Peak concentration = 1,000,000,000 gc/g-wet, 158,490,000 gc/g-wet, and 100,000,000 gc/g-wet for JN.1, BA.2.86, and ‘other Omicron variants’ respectively	Conducted in Japan. BA.2.86 and JN.1 had significantly higher shedding rates compared to the temporally or phylogenetically preceding Omicron variants, which was confirmed by a Tukey–Kramer post hoc analysis (p = 0.0010)

*Note:* Study [35] used dry weight, while the other two studies [7, 20] used wet weight. To harmonize the units, we converted dry to wet weight based on a wet mass of 128 g/person/day, corresponding to a dry mass of 29 g/person/day [59].

Numerous studies have examined the temporal kinetics of virus shedding using Ct values [19, [26], [27], [29]–[31], [43], [44], [46]–49], Kaplan–Meier plots [24, [25], 42], or Weibull regressions [51]. In studies that used Ct values and therefore did not report a direct quantitatively interpretable viral load, SARS-CoV-2 RNA was found to be detectable in almost all patients in the first week after symptom onset. After the second week of symptom onset, Ct values increased rapidly, indicating an exponential decrease in viral load. Hence, these studies support results of the three studies illustrated in (Figure 5).

### Objective 3: Duration of SARS-CoV-2 virus shedding in stool

**Figure 6.**
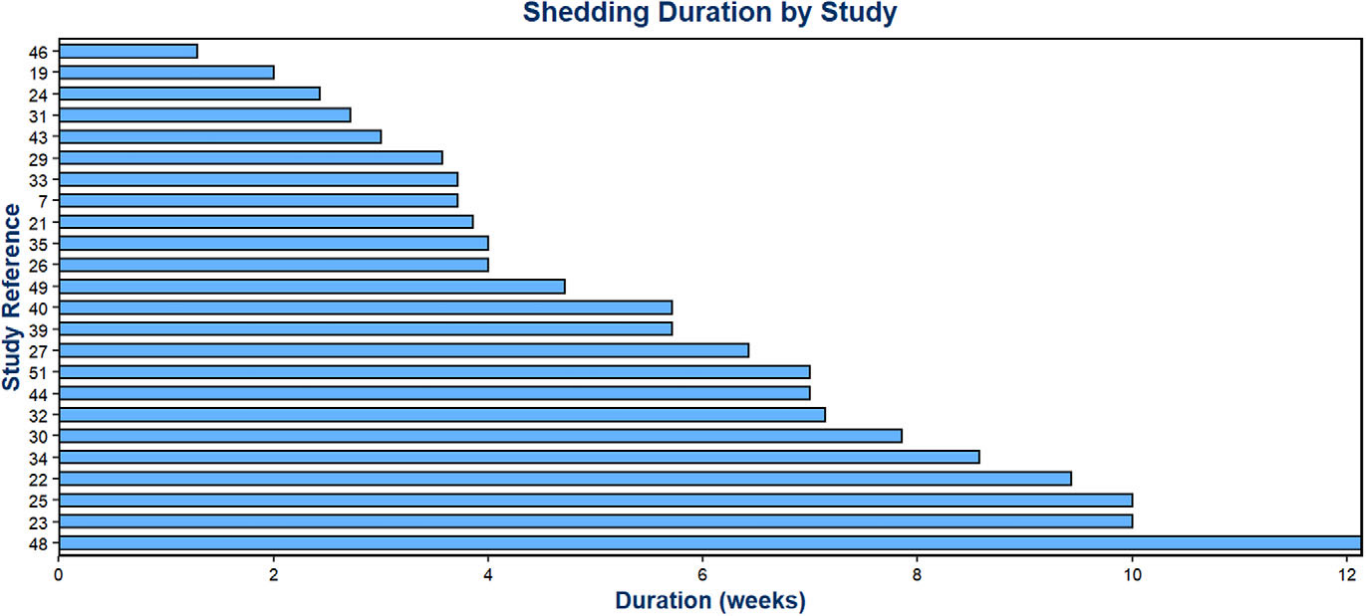
Maximum duration of SARS-CoV-2 stool shedding reported across the 24 studies.Note: one exceptional study [41] providing positivity rates up to seven follow-up months is not considered here, for more details see the ‘Results’ section.

The duration of detection of SARS-CoV-2 RNA in stool among SARS-CoV-2-infected people who shed the virus at all has been measured in several studies. Some have stopped taking faecal samples in individual patients after obtaining one or mostly two, negative samples. Several studies have followed up patients for the time period they were hospitalized, which favours obtaining samples from patients with more severe disease, whose stools were perhaps more likely to still be positive for a longer time than among patients with milder disease. Thus, most studies did not sample their entire patient cohort (or even close to the entirety) systematically for the same extended period of time. Due to limitations in the available studies, we have refrained from calculating a summary value. The included 25 studies followed between 3 and 173 patients (median = 13), (Table A3: 13 studies with systematical sampling; Table A4 (12 studies without systematic sampling), (see Appendix)). While the majority of studies reported shedding durations ranging from 2 weeks to 12 weeks (Figure 6), one exceptional study documented a considerably longer shedding period [41]. This study monitored 60 patients for 28 weeks (7 months), collecting an average of three stool samples per patient. After 7 months, SARS-CoV-2 RNA was detected in 4% of samples, but it is not clear if these patients had provided stool samples and tested positive continuously throughout the entire 7 months. Out of the 25 studies, 20 provided a direct comparison of respiratory and faecal shedding. Among those, 17 studies found that shedding in the respiratory tract stopped before faecal shedding ceased [7, [19], [21], [22], [27], [30]–[34], [36], [39]–[41], [43], [44], 51], while three studies found that shedding in the respiratory tract lasted longer than in stool [25, [29], 48].

## Discussion

This review aimed to examine the frequency, dynamics, and duration of SARS-CoV-2 RNA shedding in the stool of infected individuals. We found that approximately half of the people infected with SARS-CoV-2 shed viral RNA in the stool. While significant differences were identified between age groups, no significant differences were found when stratifying by clinical severity. We estimate that among SARS-CoV-2-infected individuals who shed SARS-CoV-2 in their stool, about half of these do so for 22 days, with a peak viral load observed during the first or early second week after symptom onset. Maximum VST is reported to range between 2 and 12 weeks.

### Proportion of persons infected with SARS-CoV2 who shed viral RNA in the stool

Our estimated proportion of SARS-CoV-2-infected persons, who shed viral RNA in their stool (54%), is comparable with findings from four systematic reviews estimating between 48 and 55% [8, 9, 11, 52], while three others reported proportions farther away (35% [10], 43% [14], and 66% [3] respectively). Differences between reviews may be attributed to variations in the inclusion criteria of the included studies. At any rate, the studies included in our review are remarkably heterogeneous, stemming from different study designs, patient populations, and laboratory methods used during the data collection process. Moreover, the proportion of SARS-CoV-2-infected persons shedding viral RNA in the stool seems to be associated with the size of the study. An obvious observation is the higher proportion among the smaller studies, which may reflect publication bias, because studies with negative findings of SARS-CoV-2 in the stool might be published less often.

Further stratification by clinical severity revealed detection rates of 35% among asymptomatic individuals, 39% among symptomatic non-hospitalized individuals, 39% among hospitalized individuals with mild to moderate symptoms, and 42% among those hospitalized with severe illness. These differences were not statistically significant (chi-square test, p-value = 0.49), suggesting no clear link between symptom severity and stool-based viral RNA detection. A meta-analysis reported a higher proportion of faecal RNA detection in patients with more severe disease (60% vs. 47% for mild or moderate cases), although the between-group differences were also not statistically significant [53]. Similarly, another meta-analysis reported no significant difference in faecal RNA positivity between severe and non-severe cases (odds ratio = 2.009, p-value = 0.079) [54].

The majority of included studies in our review were based in clinical or hospital settings, which likely overrepresent individuals with more severe disease. Asymptomatic or mildly symptomatic individuals – who may not seek medical care or be routinely tested – are likely underrepresented.

While the lack of significant association in our findings does not rule out the possibility of a relationship between severity and viral shedding, factors such as the timing of sample collection could influence detection rates. For example, hospitalized patients may have their samples collected later in their illness, potentially leading to an underestimation of shedding proportion.

Although we did not conduct a formal meta-analysis or funnel plot to assess publication bias, it is important to note that studies with low or negative detection rates may have been less likely to be submitted or accepted for publication, potentially inflating the overall PR estimates. Future studies might recruit participants using community-based strategies, such as targeting households with a positive primary case, to obtain more accurate estimates with minimal bias and assess the true burden of stool-based viral RNA shedding across different clinical presentations.

The proportion of individuals with detectable SARS-CoV-2 RNA in stool was higher in children (61%) than in adults (53%; p = 0.02). Although two systematic reviews reported higher detection rates in children, they concluded that the difference was not substantial [10, 14]. Three other systematic reviews and meta-analyses found considerably higher detection rates in children (86–89%) [55–57], compared with 54% in adults [4], although statistical significance was not discussed. This finding is noteworthy, as children have been reported to shed significantly less SARS-CoV-2 in respiratory samples compared with adults [50, 58]. It remains unclear why viral traces cannot be detected in stool samples from many infected individuals. Possible reasons include the sensitivity of the detection methods, timing of collection of the stool sample, prevailing immunity at infection, or biological variation among infected hosts.

### Viral shedding dynamics: (a) positivity rate over time, after symptom onset

Our results indicate that half of the people who shed viral RNA in the stool will do so for about 22 days, which equals the median clearance reported in a previous systematic review [10]. Additionally, these findings align with another early systematic review by Morone et al. (2020) with a somewhat shorter median duration of 19 days [52]. However, in contrast to our review, Morone et al. included more than 25 case reports of patients with frequently mild illness and/or follow-up periods that were not long enough to meet the inclusion criteria of at least two weeks in our review. Both aspects may have led to a slightly shorter PR over time compared with our study.

In general, a common bias in larger studies is the focus on hospitalized patients, leading to an overrepresentation of more severe cases who stay in the hospital for a longer time and might be more likely to shed the virus longer in stool. Upon preparation of a review, such studies may potentially lead to a certain overestimation of shedding duration.

### Viral shedding dynamics: (b) quantity (viral load) over time (kinetics)

Due to the limited number of robust studies, only three studies were suitable to analyse the (quantitative) kinetics of the viral load [7, [20], 35]. Results suggest that viral shedding may peak during the first or early second week after symptom onset. This pattern applies to symptomatic individuals, as asymptomatic cases lack, by definition, a defined onset of symptoms. The observed time lag between peak shedding in the URT and stool – ranging from several days to about one week – may reflect the time needed for the virus to be swallowed, perhaps infect intestinal cells, and be excreted (Figure 5).

The three studies were conducted at different points in the pandemic, capturing periods with different dominant SARS-CoV-2 variants. We report normalized values because the absolute quantities of viral load cannot be easily compared across variants due to differences in methodology and measurement. Notably, the duration of sample collection in one study spanned several consecutive periods with different dominant Omicron variants [20]. The authors noted that a significantly higher number of genome copies were measured for the BA.2 and JN.1 variants compared with those that preceded them chronologically or phylogenetically.

These findings have direct relevance for wastewater-based surveillance. The continued detectability of viral RNA in stool – despite vaccination, prior infections, and viral evolution – indicates that wastewater monitoring remains a feasible and informative tool. In addition, understanding shedding kinetics helps interpret wastewater signals more accurately – for example, in differentiating between ongoing transmission (incidence) and residual signal from prolonged shedding (prevalence). Clarifying this distinction is critical for accurate public health interpretation. Together, these insights support the continued value of wastewater surveillance in tracking SARS-CoV-2.

### Duration of SARS-CoV-2 virus shedding in stool

Our review also investigated the maximum duration of SARS-CoV-2 viral shedding in stool across the included studies. Again, study methodologies varied widely. For many study investigators, systematic follow-up was a challenge – for example, when hospitalized patients were clinically fit for discharge but may still have had detectable viral RNA in their stool. Although many studies applied a systematic sampling approach – often defining the end of shedding after two consecutive negative stool samples – several others predefined a fixed period for sample collection regardless of patient shedding status. This design choice may have led to truncation of the actual shedding duration. For instance, Young et al. collected stool samples only during the first two weeks following study enrolment, with the maximum reported VST being 9 days [46]. Lo et al. followed participants for one month with a maximum VST of 19 days [31], while Vaselli et al. limited collection to three weeks, reporting a maximum VST of 21 days [43]. These predefined periods may have missed longer shedding durations. Nevertheless, it is noteworthy that two studies using a systematic sampling approach – defining the end of shedding after two consecutive negative stool samples – still reported relatively short maximum durations of shedding: 14 days [19] and 17 days [24]. Hence, because of the methodological differences, the range of reported maximum durations of faecal shedding (2–12 weeks) is not surprising. Interestingly, one study detected SARS-CoV-2 RNA in 4% of stool samples after 28 weeks [41]. Compared with the other studies, this duration is exceptionally long. In this study, an average of 3 stool samples per patient suggests that patients were not closely followed up. Without longitudinal follow-up of the same patients, it is difficult to determine whether the detection of SARS-CoV-2 RNA in stool after such a long time reflects continuous shedding from a single infection or another infection acquired during the study period. Whole-genome sequencing might have helped in validating the results. Regarding the question of the sequence of respiratory and faecal shedding, there seems to be a consensus that viral shedding in stool starts later and lasts longer than in the respiratory tract.

This review has a number of limitations. These include the limited generalizability of our findings due to heterogeneity among the included studies and likely a substantial publication bias. We did not account for several factors that could be related to faecal viral shedding, such as demographic variables (e.g., sex and ethnicity), pre-existing conditions, and differences between symptomatic and asymptomatic individuals. We did take the vaccination status into consideration; however, as the vast majority of studies were conducted in 2020, vaccination was not an issue, as vaccines were not available yet in 2020. Given the limited availability of high-quality studies, conclusive statements regarding differences in shedding kinetics among SARS-CoV-2 variants cannot be drawn.

For the research questions explored in this review, we propose a number of recommendations to enhance the quality of future studies (Table 4).

**Table 4.** Recommendations for enhancing the quality of future studies on SARS-CoV-2 stool shedding

Recommendation	Details
1. Pre-planned data collection	Follow a pre-planned protocol for data collection.
2. Proportion of SARS-CoV–2-positive individuals with viral RNA in stool	Multiple stool samples should be collected, and sampling should start within the first week of illness. Important co-factors, such as age, sex, and disease severity, should be documented and reported when possible.
3. Shedding dynamics (PRs over time/kinetics)	Samples should be collected systematically, ideally at consistent intervals and over a uniform period for all participants, irrespective of the clinical course. Contextualization regarding the day post-symptom onset, along with the provision of quantitative values (e.g., copies of viral genome per gram of stool rather than Ct values), will facilitate comparisons between different studies. Negative stool samples must be included in the analysis of median shedding values. Additionally, discontinuing sampling after multiple negative results should be incorporated into the analysis (as continuously negative) to ensure biologically plausible curve trajectories and to exclude implausible patterns, such as a resurgence of viral load two weeks after symptom onset. While a larger sample size would allow for more precise estimates, the aforementioned factors are of greater importance.
4. Maximum duration of shedding	Samples must be systematically collected over an extended period. Furthermore, the approach for discontinuing sampling after multiple negative results should be clearly documented and incorporated into the analysis.

## Conclusions

Our study suggests that viral shedding of SARS-CoV-2 can be detected in stool samples of approximately 50% of infected individuals. Among those with detectable viral shedding, the PR decreases to about 50% after three weeks post-symptom onset, with viral load peaking between days 3 and 9 after symptom onset. The maximum viral shedding duration may last up to 12 weeks. Methodological limitations, such as non-systematic data collection, unclear methodologies, and inadequate reporting of results, hinder the drawing of strong and reliable conclusions. The implementation of more standardized methods would enhance the robustness and comparability of findings.

## Supporting information

10.1017/S0950268826101241.sm001 Abunijela et al. supplementary material Abunijela et al. supplementary material

## Data Availability

The data from the scoping review are provided in the additional file. Further inquiries can be directed to the corresponding author.

## Abbreviations

CI: confidence interval
gc/g-wet: RNA gene copies per gram of wet stool
PR: positivity rate
VST: viral shedding time

## Supplementary material

The supplementary material for this article can be found at http://doi.org/10.1017/S0950268826101241.
